# Efficacy of Patellar Taping and Electromyographic Biofeedback Training at Various Knee Angles on Quadriceps Strength and Functional Performance in Young Adult Male Athletes with Patellofemoral Pain Syndrome: A Randomized Controlled Trial

**DOI:** 10.1155/2022/8717932

**Published:** 2022-08-01

**Authors:** Shahnaz Hasan, Asma Alonazi, Shahnawaz Anwer, Azfar Jamal, Suhel Parvez, Faiz Abdulaziz Saleh Alfaiz, Heng Li

**Affiliations:** ^1^Department of Physiotherapy, College of Applied Medical Sciences, Majmaah University, Al Majma'ah, Saudi Arabia; ^2^Department of Building and Real Estate, The Hong Kong Polytechnic University, Hung Hom, Hong Kong, China; ^3^Health and Basic Science Research Centre, Majmaah University, Al Majma'ah 11952, Saudi Arabia; ^4^Department of Biology, College of Science, Majmaah University, Al Majma'ah 11952, Al-Zulfi, Riyadh Region, Saudi Arabia; ^5^Department of Medical Elementology and Toxicology, School of Chemical and Life Sciences, Jamia Hamdard, New Delhi 110062, India; ^6^College of Science, Majmaah University, Al Majma'ah 11952, Al-Zulfi, Riyadh Region, Saudi Arabia

## Abstract

**Background:**

The severity of the articular lesion is the single most essential element in investigating the extent of flexion that is required for activities. However, a prior study found no differences in muscle strength gains of quadriceps muscles at different knee angles in people with patellofemoral pain syndrome (PFPS).

**Objective:**

The effects of patellar taping and electromyographic biofeedback (EMG-BF)-guided isometric quadriceps strengthening at different knee angles (e.g., 30°, 60°, and 90° of knee flexion) on quadriceps strength and functional performance in people with PFPS were compared in this single-blind randomized controlled parallel trial.

**Methods:**

Sixty adult male athletes with PFPS (age: 26.9 ± 1.4 years) were randomly divided into two groups. The experimental group (*n* = 30) received patellar taping and EMG-BF-guided isometric contraction exercise at 30°, 60°, and 90° angles, and the control group (*n* = 30) received sham patellar taping without EMG-BF-guided exercises for six weeks. Pain intensity, knee function, muscle strength, and the single-leg triple hop (SLTH) test were assessed.

**Results:**

The pain intensity and SLTH scores between the groups were significantly different at the end of the trial (*p* ≤ 0.001). The EMG-BF and control groups had mean pain scores of 1.3 (0.8) and 4.5 (0.8), respectively. The EMG-BF and control groups had mean functional scores of 80.4 (5.1) and 69.1 (6.1), respectively. The mean SLTH score for the EMG-BF group was 540.7 (51.2) and for the control group it was 509.4 (49.8) after the trial. Quadriceps muscle strength was significantly higher in those who performed quadriceps strength training at 60° of knee flexion after six weeks than in those who performed strength training at 30° or 90° of knee flexion.

**Conclusion:**

The findings indicated that individuals who trained their quadriceps at a 60° knee angle had significantly stronger quadriceps muscles than individuals who trained at 30° or 90° of knee flexion. *Trial Registration*. This trial is registered at Clinical Trials.gov under the identifier NCT05055284.

## 1. Introduction

Patellofemoral pain syndrome (PFPS) is referred to as peripatellar or retropatellar pain, which is characterized by alterations in the physical and biomechanical features of the patellofemoral joint [1]. The most excruciating discomfort is experienced when sitting or kneeling for extended periods of time with bent knees and climbing or descending stairs. The PFPS is the leading cause of knee discomfort in young adults [2, 3]. It is also one of the most prevalent knee injuries reported in athletes engaging in a wide variety of sports [4], with a frequency of roughly 30% and accounting for 9% of all injuries sustained by young athletes [5–7]. A refractory and benign-sounding theory about PFPS has been disproved [8]. Current medical research claims that the condition is more dangerous and long-lasting and is linked to early degenerative changes in young people with anterior knee pain [9, 10]. In the event that this occurs, certain young patients diagnosed with PFPS may be at a higher risk of injury to their anterior cruciate ligament (ACL) [11].

Even though PFPS is a widespread issue, there is no agreement about its causes, diagnostic criteria, and treatment options [12]. Patellofemoral pain has been attributed to a variety of factors [7]. The hypothesis that certain modifiable risk factors, such as vastus medialis obliquus (VMO) weakness, patellar hypermobility, and patellar malalignment, help explain the development and recurrence of PFPS has been made [13, 14]. One of the crucial parameters that can help explain why people develop PFPS is quadriceps muscle strength, which has a strong correlation with the condition [15–17]. The evidence indicates that patients with PFPS have a weaker quadriceps muscle than healthy individuals [17]. Injury to the quadriceps, whether it be through inhibition or atrophy, can lead to a reduction in muscle peak torque [15]. This reduction in muscle peak torque is one of the potential causes of PFPS [15]. Patellar tilt is a condition that can be made worse by muscular imbalances in the quadriceps heads, particularly in the vastus medialis oblique (VMO) and vastus lateralis (VL) muscles [18]. Studies show that strong VMO muscles play a vital role in combating PFPS by keeping the patella stable [18, 19]. Delayed and insufficient VMO activation results in knee maltracking (which is also called “patella maltracking”), causing the knee joint to not function properly [20].

There are numerous contributing factors to PFPS symptoms, making treatment difficult. Stretching the tight lateral structure and general quadriceps strength training are both included in treatment programs because they are thought to stimulate VMO activation [21, 22]. PFPS treatment typically includes patient education, electromyographic biofeedback, activity adjustment, neuromuscular electric stimulation (NMES), knee braces and orthotics, physical agents for deep and superficial heating, and nonsteroidal anti-inflammatory medications [23]. In order to concentrate the patella and improve patellar tracking, it was hypothesized that patella taping would generate a mechanical medial shift. When done correctly, patellar taping can help alleviate pain associated with activities that generate significant patellofemoral joint reaction forces [24]. Also, taping can help alleviate some of the short-term pain associated with exercise [25].

The mechanics of the patellofemoral joint may also be affected by strengthening of the quadriceps, particularly the VMO. The medial movement of the patella can only be achieved by the VMO, which is also the only muscle that is active throughout the whole range of motion [26]. In addition, past studies have indicated that combining regular quadriceps exercises with electromyographic biofeedback (EMG-BF) training can help persons who have PFPS improve their symptoms as well as their quadriceps strength [27–29]. Steinkamp et al. [30] established that closed-kinetic chain exercises performed at 0 to 40 degrees of knee flexion reduce patellofemoral joint reaction forces. Due to the reduced patellofemoral joint response forces and strains, individuals with PFPS may be better managed by a closed-kinetic chain training program with knee flexion of 0 to 40 degrees than an open-kinetic chain exercise program [30]. Additionally, patellofemoral joint stress is increased when performing leg-press exercises at 60° to 90° of knee flexion [30]. This is especially important for people with lesions farther away from the patella, because they will need to bend their knees more to relieve stress on the distal patella [31].

Several research works on PFPS have compared typical open and closed-kinetic chain exercise programs with and without EMG-BF in an effort to determine whether or not selective activation of the VMO confers any benefits [27, 28, 32]. According to the findings of Dursun et al. [28], there was no discernible difference in the clinical improvement of the two groups of patients after three months. Similarly, Yip and Ng [32] found no statistical changes in clinical outcomes at two-month follow-up, although they suggested that biofeedback could speed healing. In contrast, Ng et al. [27] found that biofeedback groups had better VMO/VL ratios. Only a few studies have looked at how angle-specific strength training impacts quadriceps muscles in patients with PFPS, despite the large number of studies looking at quadriceps' strength at different knee angles in healthy adults. For instance, in a prior study, it was determined that maximal quadriceps torque occurs at 60 degrees of flexion (mid-range) in both male and female college students [33]. In addition, Suter and Herzog [34] and Chan et al. [35] discovered that the knee extensor torque was highest when the knee was bent at a 90-degree angle in 10 and 17 healthy individuals, respectively. Similarly, another study discovered that isometric strengthening exercises done at 90 degrees of knee flexion improved quadriceps muscular strength more than training at 45 degrees of knee flexion [36]. In contrast, Honarpishe et al. [37] revealed no differences in gain in muscle strength of VMO and VL at different knee angles in individuals with PFPS. Moreover, there is a dearth of hard evidence to back the utilization of angle-specific quadriceps strengthening exercises as a treatment for PFPS. Therefore, the purpose of this study was to compare the effects of patellar taping and EMG-BF-guided isometric quadriceps strengthening at different knee angles (e.g., 30, 60, or 90 degrees of knee flexion) on quadriceps strength and functional performance in young adult male athletes with PFPS. An angle-specific quadriceps strength change was hypothesized in this investigation. Specifically, people who practiced quadriceps strengthening at 60 degrees of knee angle were expected to gain greater strength than those who trained at 30 or 90 degrees of knee angle. The null hypothesis of this study stated that quadriceps strength does not alter depending on knee angle. This is the first study of its kind to investigate the effects of patellar taping and EMG-BF-guided isometric quadriceps training at different knee angles on functional performance and quadriceps strength in young adult male athletes with PFPS. The findings of this research could contribute to the development of an efficient method for increasing quadriceps strength as a treatment for PFPS.

## 2. Materials and Methods

### 2.1. Trial Design

**Figure 1:**
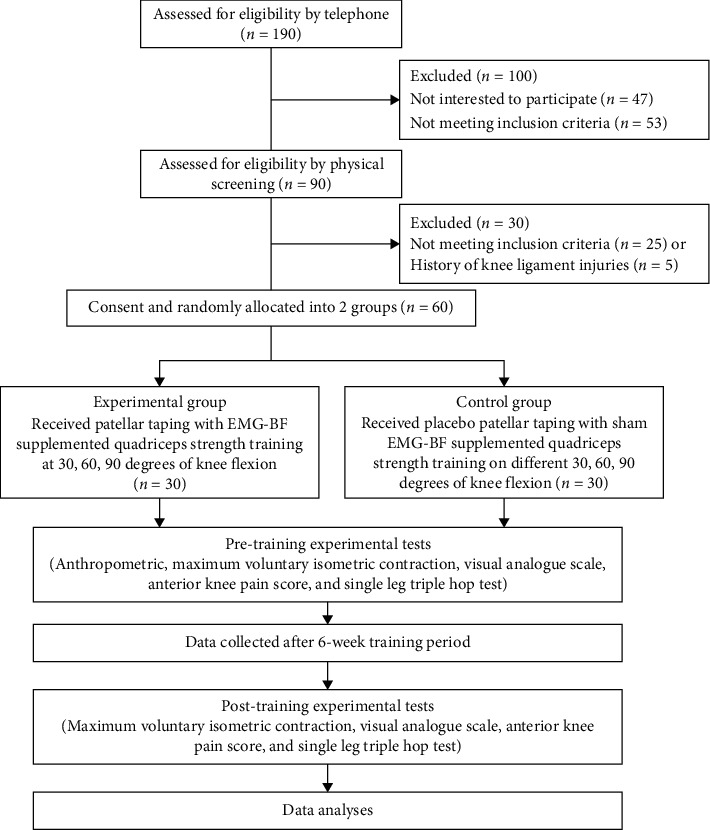
Participants' flow through each stage of a randomized trial Consolidated Standards of Reporting Trials (CONSORT) diagram.

A single-blind randomized controlled parallel trial with a 6-week intervention period was designed to evaluate the hypothesis (Figure 1). The research was carried out at Majmaah University's Rehabilitation Center in Al Majmaah, Riyadh, Saudi Arabia, from November 30th, 2020, to May 25th, 2021. A physiotherapist who specializes in musculoskeletal disorders in sports and has more than 20 years of experience conducted screenings of adult men with PFPS. They were recruited from the university's physical therapy clinic, athletics clubs, and the general public. Readings were taken before and after the test. The trial session was completed by all patients in each group. Group A (experimental group) received patellar taping with EMG-BF-guided maximum voluntary isometric contraction (MVIC) exercise at 30-, 60-, and 90-degree angles. Group B (control group) received sham patellar taping without EMG-BF-guided MVIC exercise at 30-, 60-, and 90-degree angles. The MVIC of the quadriceps muscle at angles of 30, 60, and 90 degrees, pain intensity, and functional status were the outcome measures for this study.

### 2.2. Participants

190 athletes with knee pain were evaluated over the phone. A total of 60 adult male athletes with PFPS participated in and completed the trail. The average age, height, weight, and body mass index (BMI) were 26.9 ± 1.4 years, 69.2 ± 2.01 kg, 166.8 ± 1.5 cm, and 24.8 ± 0.68 kg/m^2^, respectively. All of the participants had knee pain that had been present for at least eight weeks and was made worse by activities such as descending and ascending stairs, squatting, and running. They also had to have a positive J sign (lateral tilt of patella), a more symptomatic and malaligned knee included in the case of bilateral involvement, as well as radiographic evidence of patellar malalignment. Participants who had a history of knee fractures, patella dislocations, knee deformities (such as genu varum), flexion contractures, ligament/meniscal injuries, knee osteoarthritis, or the use of NSAIDs or intra-articular injections were not allowed to participate in the study. This study's methodology was approved by the institutional review board for research at the College of Applied Medical Science, Majmaah University, Saudi Arabia (ethics number: MUREC-Nov./COM-2O20/11-2), and it was registered at Clinical Trials.gov under the identifier NCT05055284. Participants in the study are provided with information regarding the potential disadvantages and advantages of taking part in the investigation, and they are also required to sign a written informed consent form in accordance with the principles outlined in the Helsinki Declaration.

### 2.3. Outcomes

#### 2.3.1. Quadriceps Muscle Strength (Primary)

**Figure 2:**
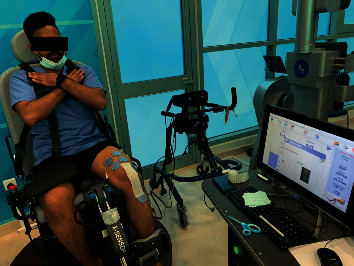
Participants' position during quadriceps strength measurement.

A valid and reliable ISOMOVE dynamometer (ISO-MANSW-IT, TecnoBody, Dalmine, Bergamo, Italy) was used to measure the quadriceps femoris muscle strength at 30, 60, and 90 degrees of knee angle. Software version 0.0.1 of the ISOMOVE system (ISO-MANSW-IT, TecnoBody, Dalmine, Bergamo, Italy) was employed to collect all the data. Subjects were given an orientation with respect to the tools. Baseline (before the treatment) and posttreatment muscle strength at the end of 6 weeks were recorded. The participants were kept in position by using safety belts across their chests, thighs, and hips. Additionally, the shin pads were modified so that they were 5.1 centimeters (about 2 inches) higher than the medial malleolus (as shown in Figure 2). The testing was performed with the participant's dominant leg at 30-, 60-, and 90-degree flexion at the knee. While participants follow the verbal instructions and encouragement to keep their arms crossed over their chests to achieve maximum effort during the 5-second contractions, verbal instructions and encouragement are also given to help the participant perform the task. For each test, three consecutive trials were conducted with 2 minutes of rest in between. For the purposes of the statistical analysis, the mean score was used.

#### 2.3.2. Pain Intensity (Secondary)

A visual analogue scale (VAS) was utilized in order to quantify the level of discomfort being experienced. Knee pain can be evaluated using this scale, which has been shown to be reliable and valid [38]. On a scale from 0 to 10, the participants' current levels of pain were rated by the researchers, where 10 indicates the maximum pain and 0 means no pain.

#### 2.3.3. Knee Function (Secondary)

A validated version of the Kujala Anterior Knee Pain scale was utilized in order to evaluate knee function [39, 40]. It has 13 questions to help determine various problems of PFPS, including walking, squatting, stair climbing, jumping, running, pain, and abnormal or painful kneecap movement. The total score might be anywhere between 0 and 100. The functional capacity is more accurately reflected by a higher score. Before being administered to patients who suffered from patellofemoral pain, the Kujala questionnaires were first translated into Arabic and then checked by individuals who were fluent in the language. The translators of the Kujala questionnaire all came from a medical background and had an extensive amount of expertise regarding the primary source.

#### 2.3.4. Single-Leg Triple Hop (SLTH) Test (Secondary)

**Figure 3:**
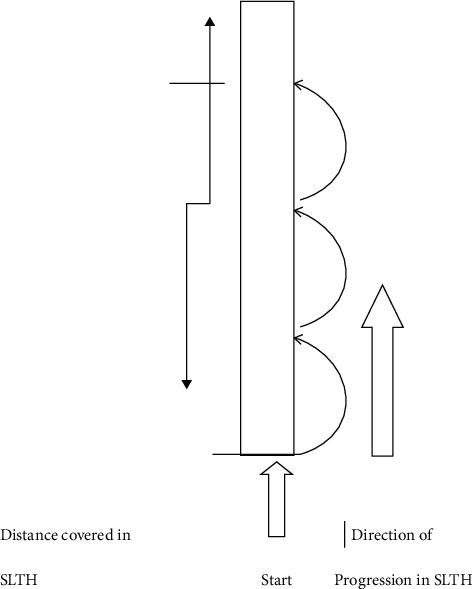
Illustration of single-leg triple hop (SLTH) test.

The single-leg triple hop test, often known as the SLTH, is frequently utilized in clinical practice to measure the knee's dynamic stability. This test comprises both the landing phase and the propulsion phase [41]. Researchers believe the hop test may be an effective screening tool for those at risk of knee injury as well as a means of assessing the progress of patients with PFPS and those who have undergone an ACL reconstruction [42, 43]. Additionally, to test lower extremity muscle strength, the SLTH test is employed as a measure of physical performance that requires a lot of muscular activity [44, 45]. As a result, the performance of the participants in this study was evaluated based on their results on the SLTH test, which was performed by jumping three times in a row. Participants began by standing on the dominant limb with their toes directly below the starting line and then hopping three times on the same limb. During the SLTH test, the distance that each participant travelled from the beginning point to the point where the back of their heels made contact with the ground was measured (Figure 3). They performed three separate tests, with a three-minute break in between each one. The best of the three, or the one with the most distance covered, was selected as the baseline.

### 2.4. Interventions

#### 2.4.1. EMG-Biofeedback

**Figure 4:**
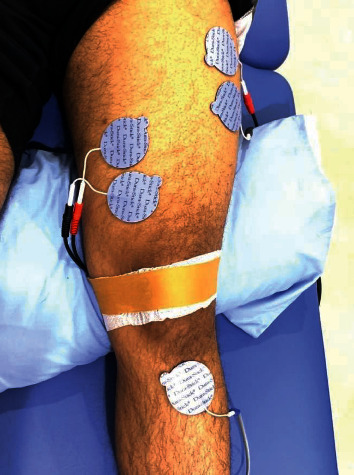
Placements of surface electrodes for electromyographic biofeedback.

An EMG-BF (Myomed 932, Enraf-Nonius, Rotterdam, Netherlands), two-channel device, was used to help participants strengthen their quadriceps muscles, while they were lying supine on a normal examination table [46–49]. For the VMO, two pairs of reusable adhesive surface electrodes were positioned spanning a distance of three centimeters medial and four centimeters proximal to the superomedial portion of the patella. Electrodes were positioned slightly downward and medially over the lower third and middle of the leg [46, 47]. This was done to stimulate the rectus femoris (RF) muscle. The active electrodes were spaced 2.1 centimeters apart, and their orientation was such that they were parallel to the direction in which the muscle fibers ran. On the proximal surface of the tibial tubercle, a reference electrode was positioned (as shown in Figure 4). Before using the surface electrodes, participants were told to shave and wash the affected area with ethanol to minimize skin resistance.

After the electrode was properly prepared and positioned, each participant was given three different exercise regimens that were to be completed with patellar tape on five days a week for a total of six weeks. To avoid the effects of tiredness, the participants were encouraged to pay attention to their muscle activity levels while exercising, to use constant verbal encouragement throughout a maximal excursion, and to rest for 10 seconds between sets. To determine the threshold level, the individual was instructed to contract the quadriceps muscle as hard as possible before each session. Training involved having participants contract their VMO and RF to a level above their threshold and holding the contraction for five seconds while an auditory signal was provided. Each participant was guided to perform three types of quadriceps exercises: isometric quadriceps, MVIC at 30°, 60°, and 90° angles, and isometric hip adduction exercises for strengthening quadriceps muscles as described below. Due to the fact that no single exercise resulted in maximal quadriceps muscle activation, this study chose a combination of exercises rather than a single exercise to maximize the possibility of recruiting maximal quadriceps muscle activity [50]. Furthermore, a previous study found that hip adduction exercise can activate the VMO more selectively, thereby balancing the VL and VMO [51]. All of the exercises were done in an exercise lab under the watch of a physical therapist.

#### 2.4.2. Quadriceps Strengthening Exercises

*(1). Isometric Quadriceps Exercise.* Participants were instructed to lie on their backs with their knees bent and a towel roll placed under their knee. Participants were told to tighten their legs by pulling on their quadriceps muscles during the exercise and keeping the audible signal on for 5 seconds during three sets of 10 repetitions.

**Figure 5:**
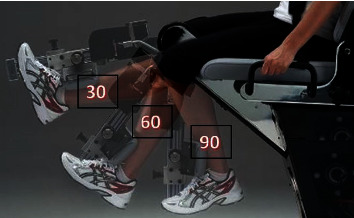
Maximum voluntary isometric contraction exercises at 30, 60, and 90 degrees of knee flexion angles.

 *(2)*. *Maximum Voluntary Isometric Contraction Exercises at 30, 60, and 90 Degrees of Knee Flexion Angles*. Participants were asked to sit on the ISOMOVE system (ISO-MANSW-IT, TecnoBody, Dalmine, Bergamo, Italy) to perform MVIC exercises of the quadriceps at 30, 60, and 90 degrees of knee flexion angles, three times per week for six weeks. The participants were instructed to perform three sets of two MVIC of quadriceps for 5 seconds each, with a 30-second rest between each set. To make sure that the MVIC exercise was done at the same knee angle every time, the participants used the ISOMOVE system (ISO-MANSW-IT, TecnoBody, Dalmine, Bergamo, Italy) to set the target knee angle before each time they did the exercise (Figure 5).

*(3). Isometric Hip Adduction Exercise.* The participants were told to lie supine with a pillow between their knees and to press the cushion between their knees to the maximum to activate the muscle via isometric hip adduction exercise. The participants were instructed to contract their muscles beyond their threshold level in order to straighten their knees, and they were to maintain that contraction for a period of five seconds in order to ensure that the audible signal was maintained throughout the exercises. The exercise was completed in three sets of ten repetitions each.

#### 2.4.3. Control Group (Sham Patellar Taping and EMG-BF-Guided Strength Training)

Participants in the control group were given the same series of exercises along with sham patellar taping and EMG-BF. When the patient's knee was flexed, a nonrigid hypoallergenic tape called placebo tape was applied in a vertical manner from the center of the patella. The electrodes were placed in a location that was remote from the muscles that were being stimulated. Below the tibial tuberosity, the ground electrode was fastened into place. The participants were given a placebo EMG-BF and nonrigid hypoallergenic tape. They were also asked to conduct quadriceps exercises without rigid patellar taping; however, they were not given specific instructions to concentrate on the recruitment of VMO and RF muscle. To ensure that the electrodes were always positioned in the same precise area during all of the sessions, a permanent marker was used to mark each electrode.

### 2.5. Sample Size

A sample size estimation was made using two groups and two-time intervals (i.e., baseline and posttest). An online sample size calculator was utilized in the calculation of the sample size (https://www.danielsoper.com/statcalc/default.aspx), which determined that 26 individuals in each group were required to detect a large effect size (Cohen's *d* = 0.80) with 0.80 statistical power and a 0.05 alpha level. This was calculated using previously published data showing a significant gain in muscle strength in the experimental group compared to the control group (Cohen's *d* > 0.80) [29]. As a result, there were 60 participants in this trial, which allowed for a 15% dropout rate.

### 2.6. Randomization and Blinding

Two-stage randomization was utilized in this investigation. In the beginning, each participant was randomly assigned to either the experimental or control group. Folders were numbered 1–60, given secret codes by an independent evaluator, and placed in a safe locker. The next folder in the file was randomly selected by an independent evaluator after a participant had consented to participate and met the eligibility requirements. In the next stage, participants were divided into three equal subgroups (*n* = 10) and randomly assigned to three different knee angles for quadriceps strength training (i.e., 30, 60, or 90 degrees). In this single-blind study, only the outcome assessor was blinded to the study group allocation.

### 2.7. Statistical Analysis

SPSS 22.0 for Windows was used to conduct the statistical analyses. The Kolmogorov-Smirnov test was used to determine the normality of the data. The independent *t*-test was used to compare basic demographics and clinical data (e.g., VAS, AKP, and SLTH). A one-way ANOVA was used to compare quadriceps muscle strength (dependent variable) between three knee angle conditions (e.g., 30, 60, and 90 degrees of knee flexion) (independent variable) for the EMG-BF and control groups. Using 2-way mixed ANOVAs, the quadriceps muscle strength (dependent variable) was compared between knee angle conditions (independent variable) and treatment groups (independent variable). For the multiple comparison of the quadriceps muscle strength variable, the *p* value was adjusted to 0.016 (0.05/3). Where appropriate, Fisher's least significant difference (LSD) post hoc testing was used for multiple pairwise comparisons.

## 3. Results

Table 1 summarizes demographic and clinical data. All 60 enrolled participants completed the trial. The EMG-BF group's mean age was 26.8 (1.4) years, while the control group's mean age was 27.2 (1.4) years. At baseline, the mean VAS score in the EMG-BF group was 7.0 (0.7) and in the control group it was 6.8 (0.7). At week 6, the mean VAS score for the EMG-BF group was 1.3 (0.8) and for the control group it was 4.5 (0.8). At week 6, there was a significant difference in pain intensity between the groups (*p* ≤ 0.001). The mean AKP score at baseline was 42.6 (6.7) in the EMG-BF group and 46.1 (7.5) in the control group. The mean AKP score at week 6 was 80.4 (5.1) in the EMG-BF group and 69.1 (6.1) in the control group. At week 6, there was a significant difference in AKP score between the groups (*p* ≤ 0.001). At baseline, the mean SLTH score was 501.3 (53.9) in the EMG-BF group and 499.9 in the control group (50.6). At week 6, the mean SLTH score was 540.7 (51.2) for the EMG-BF group and 509.4 for the control group (49.8). At week 6, a significant difference in SLTH scores was observed between the groups (*p* ≤ 0.001).

**Figure 6:**
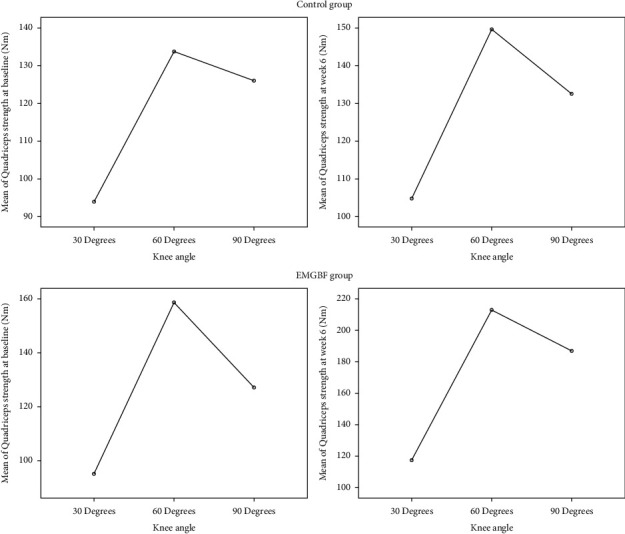
Comparison of quadriceps strength at baseline and after training.

**Figure 7:**
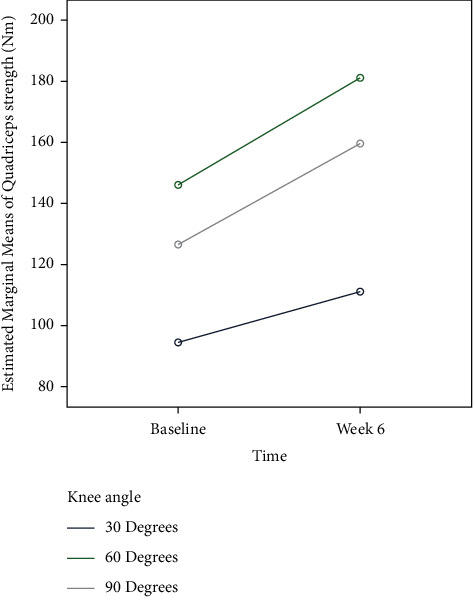
Comparison of quadriceps strength at different knee angles.

Table 2 provides a summary of the findings from one-way analyses of variance. Results revealed a significant difference in quadriceps muscle strength in individuals who trained at 30, 60, or 90 degrees of knee angle in both the EMG-BF and control groups (Figure 6). After 6 weeks, people who did quadriceps strength training with their knees bent at 60 degrees had much stronger quadriceps muscles than those who did quadriceps strength training with their knees bent at 30 or 90 degrees (Figure 7).

**Figure 8:**
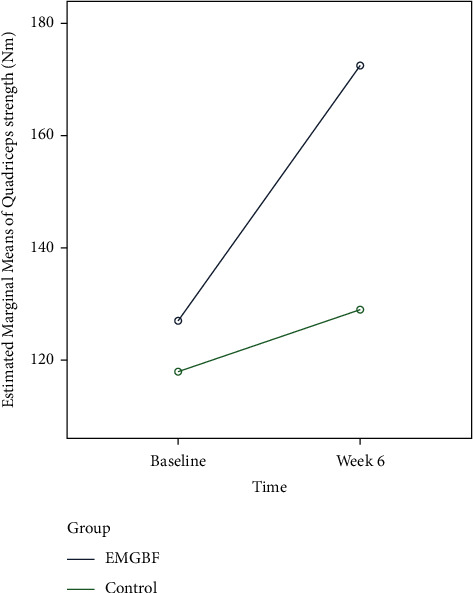
Comparison of quadriceps strength between training and control groups.

Table 3 summarizes the findings of the two-way repeated measures analysis of variance (2^*∗*^3^*∗*^2). The results indicated that quadriceps muscle strength was significantly affected by treatment group (EMG-BF versus control) (*p* ≤ 0.001), training conditions (30 versus 60 versus 90 degrees of knee flexion) (*p* ≤ 0.001), and group^*∗*^training interaction effect (*p* ≤ 0.001), indicating that quadriceps muscle strength improved following strength training at 60 degrees of knee flexion. Similarly, individuals in the EMG-BF group demonstrated significantly greater quadriceps muscle strength than those in the control group at week 6 (Figure 8). Furthermore, the comparison of effect sizes between the two groups indicated that those who conducted exercise at 60 degrees of knee angle had a considerable effect size at both baseline and end of trial (as shown in Table 4).

### 3.1. Serious Adverse Events

There were no major adverse events recorded in this experiment. A few (*n* = 4) people in the control group who had fake patellar taping felt a little bit of pain during the SLTH test.

## 4. Discussion

This study compared the effects of patellar taping and EMG-BF-guided isometric quadriceps training at various knee angles (e.g., 30 degrees, 60 degrees, or 90 degrees of knee flexion) on pain intensity, quadriceps strength, and functional performance in young adult male athletes with PFPS. The results of this study indicated that people with PFPS experienced significantly improved outcomes in terms of pain and function following six weeks of EMG-BF-guided isometric exercises and the patellar taping. At week 6, participants in the EMG-BF group demonstrated significantly greater quadriceps muscle strength than participants in the control group. Additionally, individuals who engaged in quadriceps strength training at a 60-degree knee angle demonstrated significantly greater quadriceps muscle strength than those who engaged in quadriceps strength training at 30 or 90 degrees of knee flexion. Additionally, when two groups were compared for effect sizes, those who performed exercise at 60 degrees of knee flexion had twice the effect size of those who performed exercise at 30 or 90 degrees of knee flexion at both baseline and the end of the trial. This shows the clinical significance of the results of performing quadriceps strength training at 60 degrees of knee flexion in people with PFPS.

EMG-BF has been proposed to promote preferential recruitment of the VMO in individuals with PFPS, thereby reducing lateral patellofemoral tracking. Numerous studies have examined the efficacy of EMG-BF-guided strength training in the treatment of symptomatic PFPS [27, 29, 52]. For instance, Wise et al. [52] carried out a pilot study to investigate the effects of a progressive exercise regimen that was guided by EMG and BF on those who suffered from PFPS. They came to the conclusion that persons living with PFPS would benefit from using the EMG-BF in conjunction with a graded exercise program, since it is a method that is both effective and efficient in managing symptoms. In addition, Ng et al. [27] came to the conclusion that employing an EMG-BF-guided exercise program improved the ratio of EMG activity in the VMO to that of the vastus medialis longus in persons who had PFPS. On the other hand, other research works concluded that persons with PFPS did not experience any significant therapeutic benefits with EMG-BF when compared to quadriceps exercise alone [28, 32, 46]. Also, a recent clinical guideline [53] says that EMG-BF on VMO activity should not be used to supplement knee-focused (quadriceps) exercise for PFPS.

Due to the inclusion of EMG-BF and patellar taping in this study, it was difficult to determine the effects of patellar taping on pain severity, knee function, and quadriceps muscle strength in individuals with PFPS. There have been a number of studies that looked at the usage of patellar taping and found that it may be beneficial for reducing the amount of pain that people with PFPS experienced [54–56]. The customized McConnell taping technique is an example of a typical way for minimizing pain during a functional task, such as the step-down. This technique makes use of rigid tape to reduce any combination of lateral patellar glide, tilt, and rotation in the knee [57]. In addition, people use untailored taping on the patellar glide and medial glide [58] as well as taping designed to enhance vastus muscle activation and synergy [59]. Taping the patellar tendon across the skin has been shown in multiple studies to increase proprioception by stimulating cutaneous mechanoreceptors and, as a result, boosting afferent input to the central nervous system (CNS) [3, 60, 61]. The increased afferent fiber input and neural inhibition that follow from this phenomenon are referred to as the nociceptive effect [53, 60, 61]. The recent recommendation of the formulation of a clinical practice guideline for the treatment of PFPS was made by the Academy of Orthopaedic Physical Therapy [53]. They advocate patellar taping as part of an exercise therapy treatment plan to provide immediate pain relief and improve short-term results (4 weeks). Notably, taping techniques are not effective over time or when used in conjunction with more intensive physical therapy. Additionally, they do not advocate the use of taping to improve muscle function. Overall, taping seems to help people with PFPS in the short term, but more research is needed to find out how it affects them in the long term.

The current study discovered that individuals who trained their quadriceps at a 60-degree knee angle had significantly greater quadriceps muscle strength than those who trained at 30 or 90 degrees of knee flexion. As a result of their unique anatomical traits, the three superficial quadriceps muscle segments, including VMO, are capable of producing varying muscle torque depending on knee angle, and this causes knee angle changes to have an effect on muscle fiber length excursions [62]. It was hypothesized that performing isometric exercises while varying the angle at which the knees were bent resulted in the best development in total strength [63–65]. Isometric exercise of the quadriceps in the mid-range, as discovered in another study [66], may be effective for increasing function in people who suffer from knee problems [66]. The majority of the attribution for the voluntary isometric knee extensor torque should be given to the mechanical force-length characteristics of skeletal muscle [67], although evidence suggests a neural component [34, 68]. In light of the fact that both individual muscle fiber and whole-muscle levels appear to produce their best results with a moderate force generation length, the literature supports the theory that knee extensor torque is at its greatest when seated knee movement is occurring in the middle of knee flexion [69, 70]. In another study, both men and women in college found that peak quadriceps torque happened at 60° of flexion (mid-range) [33]. In contrast to these findings, in a study involving 10 and 17 healthy adults, Suter and Herzog [34] and Chan et al. [35] found that the knee extensor torque was highest when the knee was bent at an angle of 90 degrees. Similarly, another study found that isometric strengthening exercises performed at 90 degrees of knee flexion improved quadriceps muscle strength more than 45 degrees of knee flexion training [36]. However, a direct comparison between these results is impossible due to the methodological differences and participant characteristics.

## 5. Limitations

Our research has some limitations. First, due to the low level of female participation in Saudi Arabian sports in comparison to male participation, finding female PFPS participants was challenging. Consequently, only male athletes with PFPS were included in this study. As a result of this, the findings of this study cannot be extrapolated to apply to all female athletes who suffer from PFPS. Second, there was no follow-up in this study to see if the intervention had lasting effects. The recording of muscle strength during the follow-up period could provide information regarding the sustainability of strength changes in the quadriceps. Therefore, additional research is required to determine the long-term impact of utilizing angle-specific EMG-BF to guide strength training on individuals who suffer from PFPS. Third, the individual effects of EMG-BF-guided strength training and patellar taping were not examined despite their potential importance. Therefore, people with PFPS should be studied to discover if patellar tape improves muscle recruitment patterns while exercising at different knee angles. Fourth, the study did not assess a priori power, which could restrict the validity of the current findings. Thus, more research is required to understand the association between pain, strength, and function in those who have PFPS.

## 6. Clinical Implications

The results of this investigation could have important repercussions for clinical practice. As previously stated, we discovered that quadriceps muscle strength was highest at 60 degrees of knee flexion. It is imperative that this be taken into consideration if comparing the greatest peak torque generation achieved by different approaches. This indicates that this angle may be employed for knee strength evaluation and training in patients who have PFPS if a clinician or researcher was interested in boosting the strength of the quadriceps muscle. Even if the compressive force that the patella exerts on the femoral surface may be at its peak, it is important to keep this information in mind in clinical practice when working to strengthen the quadriceps. On the other hand, training with the knee bent 90 degrees may still be better when knee pain is made worse by too much compression. EMG-BF-guided strength training can be utilized in clinical and sports medicine to encourage patients to therapy and urge them to continue their intervention time. This type of training can also be used to improve athletic performance. In the future, we recommend that comparisons be made in PFPS between activities that involve an open-kinetic chain and those that involve a closed-kinetic chain while the knee is flexed at a variety of angles. As a result, it has been suggested that, in the course of further research, the effects of EMG-BF-guided strength training for PFPS rehabilitation should be examined in greater depth, specifically focusing on the angles of the targeted training.

## 7. Conclusion

The purpose of this study was to investigate the effects of patellar taping and EMG-BF-guided isometric quadriceps training at various knee angles (such as 30, 60, or 90 degrees of knee flexion) on the severity of pain, quadriceps strength, and functional performance in young adult male athletes who had PFPS. The results of this study suggested that, after six weeks of EMG-BF-guided isometric quadriceps training with patellar tape, persons with PFPS saw a significant reduction in discomfort as well as an increase in their ability to function. Additionally, the EMG-BF group had considerably greater quadriceps muscle strength than the control group. Also, people who trained their quadriceps with their knees bent at a 60-degree angle had much stronger quadriceps than those who trained with their knees bent at 30 or 90 degrees.

## 8. Disclosure

The funders had no role in the design of the study; in the collection, analyses, or interpretation of the data; in the writing of the manuscript; or in the decision to publish the results.

## Figures and Tables

**Table 1:** Demographics details.

Variables	EMG-BF group (*n* = 30)	Control group (*n* = 30)	Independent *t*-test		95% CI of differences	
	Mean (SD)	Mean (SD)	*t*	*p*	Lower	Upper
Age, years	26.8 (1.4)	27.2 (1.4)	−1.890	0.060	−0.818	0.018
Height, cm	1.7 (0.1)	1.7 (0.2)	1.891	0.060	−0.00019	0.00885
Weight, kg	69.4 (2.1)	68.9 (2.0)	1.329	0.186	−0.194	0.994
BMI, kg/m^2^	24.8 (0.7)	24.8 (0.7)	−0.523	0.602	−0.2547	0.1480
Visual analogue scale (VAS), 0–10 cm						
Baseline	7.0 (0.7)	6.8 (0.7)	2.206	0.029	0.025	0.442
Posttest	1.3 (0.8)	4.5 (0.8)	−27.579	≤0.001	−3.518	−3.048
Anterior knee pain score (AKP), 0–100						
Baseline	42.6 (6.7)	46.1 (7.5)	−3.333	0.001	−5.625	−1.442
Posttest	80.4 (5.1)	69.1 (6.1)	13.572	≤0.001	9.657	12.943
Single-leg triple hop test (SLTH)						
Baseline	501.3 (53.9)	499.9 (50.6)	0.180	0.858	−13.969	16.769
Posttest	540.7 (51.2)	509.4 (49.8)	4.165	≤0.001	16.505	46.228

*Note*. BMI, body mass index; SD, standard deviation; EMG-BF, electromyographic biofeedback; CI: confidence interval.

**Table 2:** Summary of the results of one-way analyses of variance.

Variable	EMG-BF			ANOVA		Control			ANOVA	
	30 degrees (*n* = 10)	60 degrees (*n* = 10)	90 degrees (*n* = 10)	*F*	*p*	30 degrees (*n* = 10)	60 degrees (*n* = 10)	90 degrees (*n* = 10)	*F*	*p*
Quadricep strength at baseline (Nm)	95.1 (10.7)	158.6 (12.5)^‡^	127.2 (16.5)^†^	167.410	≤0.001	93.9 (13.9)	133.7 (12.1)^‡^	126.0 (13.5)^†^	76.685	≤0.001
Quadricep strength at week 6 (Nm)	117.5 (14.2)	212.9 (16.2)^‡^	186.9 (24.8)^†^	203.517	≤0.001	104.8 (13.2)	149.6 (15.3)^‡^	132.5 (16.3)^†^	68.051	≤0.001

*Note*. ^‡^Significantly better than 30 and 90 degrees of knee angle group. ^†^Significantly better than 30 degrees of knee angle group. Nm, Newton-meter.

**Table 3:** Summary of the results of two-way (2 × 3) repeated measures analyses of variance.

Dependent variable	Source	Df	Partial *n*^2^	*F*	*p*
Quadriceps muscle strength (Nm)	Group (EMG-BF vs. control)	1	0.467	152.743	≤0.001
	Conditions (30 vs. 60 vs. 90 degrees of knee flexion)	2	0.765	283.021	≤0.001
	Group^*∗*^conditions	2	0.228	25.655	≤0.001

*Note*. Nm, Newton-meter; EMG-BF, electromyographic biofeedback.

**Table 4:** Summary of effect sizes (ES) between two groups.

Variable	Knee angle (degrees)	EMG-BF	Control	% difference	Es, Cohen's *d*
Quadricep strength at baseline (Nm)	30	95.1 (10.7)	93.9 (13.9)	1.27%	0.11
	60	158.6 (12.5)	133.7 (12.1)	17.04%	2.02
	90	127.2 (16.5)	126.0 (13.5)	0.95%	0.08

Quadricep strength at week 6 (Nm)	30	117.5 (14.2)	104.8 (13.2)	11.43%	0.93
	60	212.9 (16.2)	149.6 (15.3)	34.92%	4.02
	90	186.9 (24.8)	132.5 (16.3)	34.06%	2.59

## Data Availability

Data are accessible upon request from the first author.
